# Evaluation of the thoracic epidural spread after caudal thoracic contrast medium injections in cat cadavers

**DOI:** 10.3389/fvets.2025.1632175

**Published:** 2025-08-05

**Authors:** Paolo Franci, Francesco Santoro, Alessandra Landi, Luca Manassero, Elena Lardone

**Affiliations:** ^1^Department of Veterinary Sciences, University of Turin, Grugliasco, Italy; ^2^Southfields Veterinary Specialists, Basildon, United Kingdom

**Keywords:** cat, thoracic epidural anesthesia, cadaveric study, contrast medium spread, Quincke needle

## Abstract

**Background:**

Thoracic epidural anesthesia (TEA) offers significant analgesic benefits in humans undergoing major surgery. However, this technique remains underexplored in feline medicine due to anatomical challenges and limited data on epidural drug distribution.

**Objective:**

To assess the feasibility of a paramedian approach and characterize the epidural spread of 0.3 mL/kg of contrast medium administered in three consecutive aliquots of 0.1 mL/kg at the 12th–13th thoracic vertebrae (T12–T13) level in cat cadavers.

**Methods:**

Seven refrigerated cat cadavers underwent three consecutive thoracic epidural administrations of 0.1 mL/kg of iodinated contrast medium (total dose injected 0.3 mL/kg) administered at the T12–T13 interspace using a 25 G, 25 mm Quincke needle via a paramedian technique. Computed tomography was used to evaluate longitudinal and circumferential distribution 5 min after each dose.

**Results:**

Epidural spread increased significantly with incremental dosing (longitudinal *τ* = 0.63, *p* < 0.001; circumferential *τ* = 0.58, *p* = 0.001). The percentage of longitudinal and circumferential median spread increase, when compared with the previous dose, was, respectively, 46 and 45% for D2, and 17 and 70% for D3. The initial injection preferentially spread cranially (*p* < 0.05), however, cervical segments were reached in only one case, while the sacral segments were never reached. No significant effect of gravity on circumferential spread was observed.

**Conclusion:**

This study demonstrates the feasibility of TEA via a paramedian approach in cats and provides novel insight into epidural fluid distribution patterns.

## 1 Introduction

Thoracic epidural anesthesia (TEA) provides segmental blockade of the spinal nerves supplying the thoracic wall, pleura, and cranial abdomen. In human medicine, TEA can be considered the gold standard of analgesia for cardiac, thoracic and major abdominal surgery (1). Several advantages of this technique have been described in numerous clinical and experimental studies, such as a reduction of the neuroendocrine response to surgery and associated perioperative complications (2). Moreover, the resulting sympathetic blockade has shown to have protective effects on the heart, lungs and gastrointestinal tract, as well as beneficial immunological and coagulative properties (3). TEA is therefore associated with a better outcome (i.e., faster mobilization, reduced hospital stay and improved overall recovery) in patients undergoing major surgery (4).

In veterinary medicine, a limited number of studies about TEA in dogs has been published (5, 6) whereas none have been conducted in cats. When compared to humans, the lower interest toward this technique in small animals may be related with the higher level of difficulty in its execution due to vertebral anatomical differences between species. In fact, in most domestic animals, the interspinous foramen at thoracic and lumbar level is reduced, thus making needle positioning more challenging (7).

In 2021, Tonge et al. (8) described TEA in a dog undergoing thoracotomy and lung lobectomy, which resulted in intra-operative stability of vital parameters and adequate postoperative analgesia. Evidence of the perioperative efficacy of TEA in dogs undergoing major thoracic and cranial abdominal surgery was also found in a recent study by Lardone et al. (6). Although scarce, the available literature suggests a potentially important role of TEA in small animal anesthesia.

The existing evidence on the distribution patterns of drugs injected at the thoracic epidural level in domestic species is also limited. This is especially true in cats, since all the available studies in this species focus on lumbosacral or sacrococcygeal injections (9–12). To the best of our knowledge, to date, no studies on thoracic epidural injections in cats have been reported.

The aim of this study was to investigate the feasibility of a paramedian approach for peridural injections and epidural distribution of 0.3 mL/kg of contrast medium administered in three consecutive aliquots of 0.1 mL/kg at the caudal thoracic level in cat cadavers.

## 2 Materials and methods

### 2.1 Choice of cadavers

This is a prospective cadaveric study.

Refrigerated cadavers of owned cats euthanized/dead for reasons unrelated to this study and donated (via signed owner’s informed consent) to the University of Turin (Italy) for research purpose were used.

Exclusion criteria were a long refrigeration time (>36 h), poor tissue preservation, presence of spinal abnormalities and vertebral fractures. Cadavers not refrigerated immediately after death were also excluded.

For each subject, the following data were recorded: sex, breed, weight (kg), BCS (body condition score, range 1 to 5), and occipito-sacral length (cm).

### 2.2 Preparation and positioning

After trichotomy of the dorsal thoracic region, each cadaver was positioned in left lateral recumbency on a 40 × 25 × 5 cm rigid box placed on the computed tomography (CT) scanner table. In order to facilitate the epidural injections, all the vertebral columns were kept close to the lateral edge of the box and the thoracolumbar spines were flexed by an operator by manually pulling the shoulders and the base of the tail far from the edge.

### 2.3 Epidural administration

**Figure 1:**
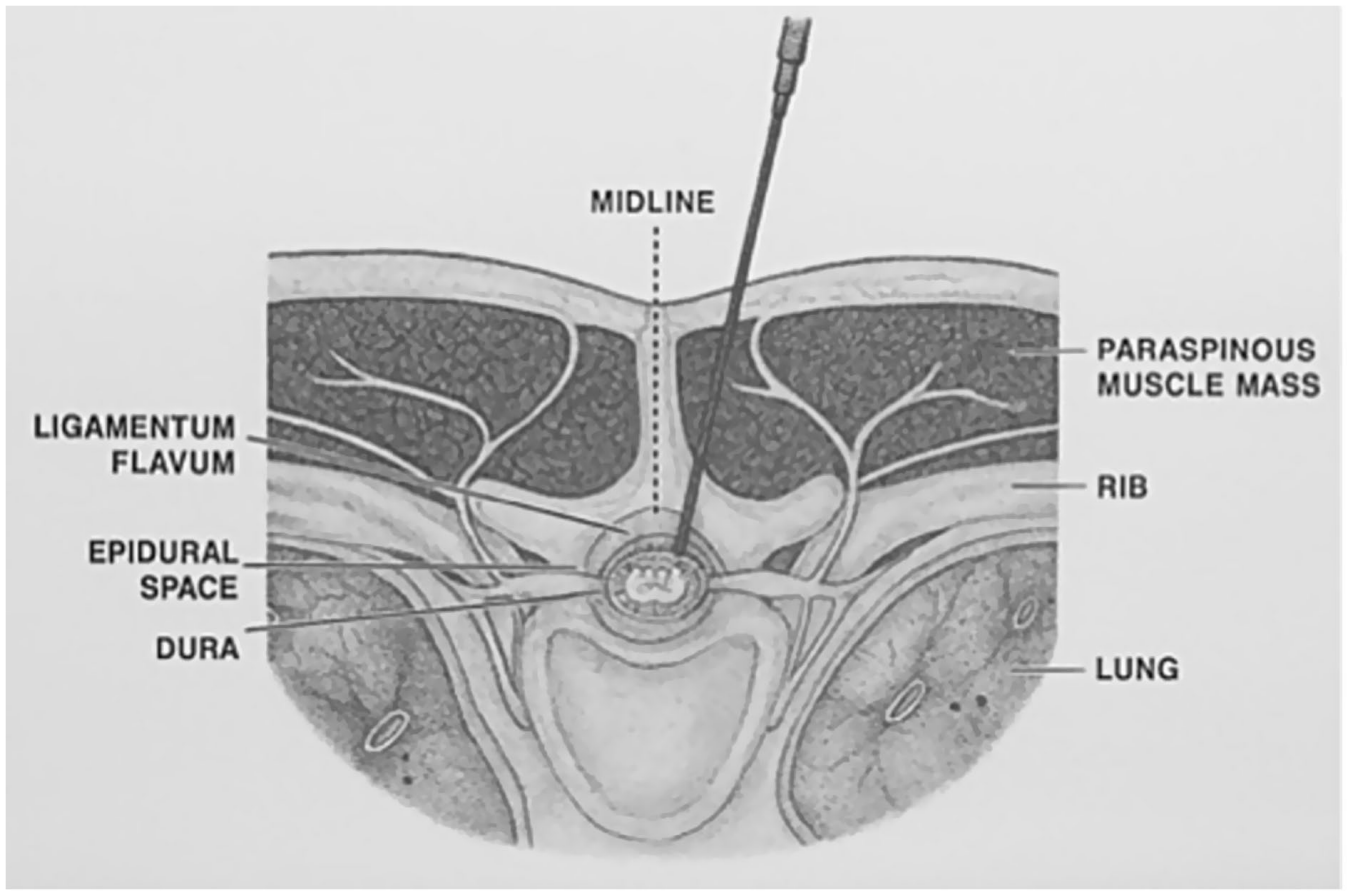
Needle insertion using a paramedian approach with a cephalad angulation.

An experienced operator (PF) inserted a 25G 25mm Quincke needle (Becton Dickinson, Italia) between 12th and 13th thoracic vertebrae (T12–T13) level using a paramedian approach with a cephalad angulation previously described in humans (13) and dogs (14). The needle was introduced at the left side of the caudal margin of the dorsal spinous process of T12 or T13 and advanced perpendicularly to the skin until it reached the vertebral lamina. The needle was then withdrawn a few millimeters and redirected in a ventral-cranial-medial direction for multiple times, until piercing of the ligamentum flavum was perceived and recorded. Correct needle positioning was ultimately confirmed by a CT scan (Figure 1).

The number of attempts needed to correctly position the needle was recorded for each cadaver. A single attempt was defined as the insertion of the spinal needle within the epidural space without any need for needle redirection, whereas any withdrawal and redirection of the needle was considered as a separate attempt. Cadavers in which an inadvertent spinal injection was observed were excluded from the study.

Once the position of the needle was deemed correct, the needle stylet was removed, and a perforable injection port was connected to the needle hub. This dead space was then primed using 1 mL of radiographic contrast medium [Iomeron (300 mg/mL; Bracco Imaging, Italy)] diluted with 3 mL of saline solution (sodium chloride, 0.9%; S.A.L.F., Italy). The same solution was administered in three consecutive aliquots (D1, D2, and D3) having an equal volume of 0.1 mL/kg based on the ideal body weight. All injections were performed at the same anatomical location—between the 12th and 13th thoracic vertebrae (T12–T13)—using a syringe connected to the injection port via a pre-filled 21G butterfly needle. To assess the extent of contrast spread, CT scans were performed 5 min after each injection, followed immediately by administration of the subsequent aliquot.

### 2.4 Measurements

The length of the needle segment extending from the cutaneous insertion point to the tip was recorded for each subject in order to quantify how deep the epidural space was from the skin.

The longitudinal spread was quantified by a trained operator (AL) by counting the number of vertebrae reached by the contrast medium at the level of the caudal margin of the intervertebral foramen. For each vertebra, the distribution pattern of the contrast medium around the spinal cord (circumferential spread) was also assessed. For this purpose, at the level of the intervertebral nerve emergence the spinal canal of each vertebra was divided in 12 clock sections, with section 6 being the pavement of the canal (most ventral aspect) and 12 its most dorsal part. The circumferential spread was therefore defined as the number of clock segments reached by the contrast medium for each vertebra. The total number of circumferential segments (12 × number of vertebrae) for the cervical, thoracic, lumbar and sacral tracts was 84, 156, 84 and 36, respectively.

**Figure 2:**
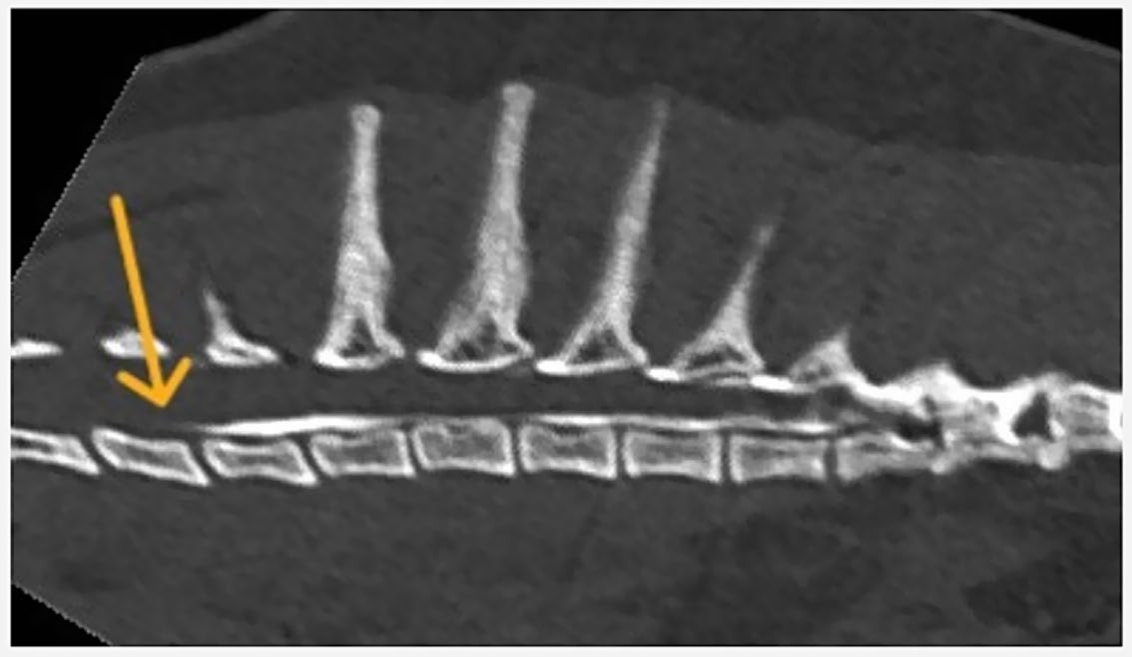
Longitudinal spread of contrast medium following injection at the T12–T13 vertebral level.

**Figure 3:**
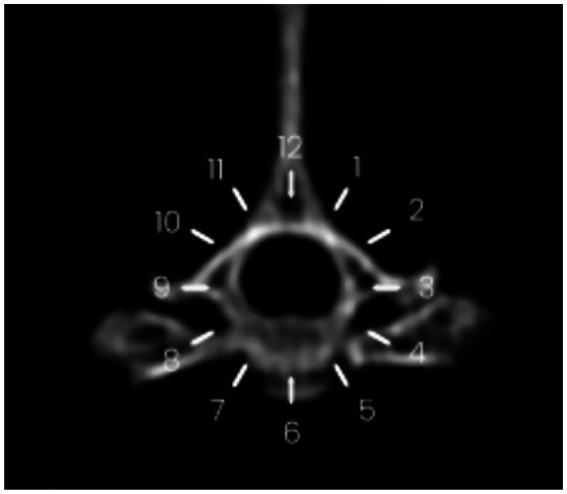
Circumferential spread of contrast medium following injection at the T12–T13 vertebral level. The spread is visualized between the 9 o’clock and 4 o’clock positions on the reference clock face.

Both longitudinal and circumferential segments were considered cranial or caudal using the injection site (space between T12 and T13) as an anatomical cutoff (Figures 2, 3).

### 2.5 Statistical analysis

Data were tested for normality using visual analysis of the distribution histograms and Q-Q plot as well as Shapiro–Wilk test.

For descriptive statistics, median was used as a measure of central tendency and dispersion was expressed as first and third quartiles [median (Q1, Q3)].

Kendall’s tau-b correlation was used to describe the relationship between the incremental doses and the total longitudinal and circumferential spread. The strength of the association was described as weak for tau-b coefficients (*τ*) < 0.2, moderate for *τ* between 0.2 and 0.3, strong for *τ* > 0.3 (15).

The difference between cranial and caudal new segments reached with each of the three doses was assessed using related-samples Wilcoxon signed rank test.

Post-hoc power calculation was calculated using G*Power (Ver 3.1.9.6). Statistical analysis and graphs design were performed using IBM SPSS Statistics (Ver. 30.0.0.0). Statistical significance was set at *p* < 0.05.

## 3 Results

The study was conducted between June 2023 and September 2024.

Nine cat cadavers were used for the study, one of which was excluded due to poor tissue preservation.

A single attempt was needed to position the needle in all the remaining 8 cadavers. One of these subjects was subsequently excluded due to an inadvertent subarachnoid injection revealed by the CT scan (success rate 87.5%). Seven adult cat cadavers were therefore included in the final spread pattern analysis.

Cadaver demographics are summarized in Table 1.

**Table 1:** Cadaver demographics.

Characteristic	
Breed	*n*
DSH	5
Persian cat	1
Chartreux cat	1
BCS	*n*/5
1	0
2	3
3	4
4	0
5	0
Occipital-sacral length	cm
m (range)	44 (39–47)
Weight	kg
m (range)	3.25 (2.3–5.5)

DSH, domestic shorthair; BCS, body condition score; m (range), median.

The median length (cm) of needle segments extending from the cutaneous insertion point to the tip was 1.33 cm (1.15–1.63).

During needle placement, the piercing of the yellow ligament was perceived in 6/7 of the subjects.

Descriptive statistics are summarized in Tables 2, 3.

**Table 2:** Longitudinal spread.

Longitudinal spread (*n* vertebrae)				
Dose	Median	95% CI	Q1–Q3	Range (min–max)
D1				
Total	14	11–16	12–15	9–19
Cranial	8	7–11	7–11	6–15
Caudal	4	3–5	3–4	3–7
D2				
Total	5	2–6	2–6	1–6
Cranial	1	0–3	1–3	0–5
Caudal	1	0–3	3–3	0–5
D3				
Total	2	1–3	1–4	0–4
Cranial	1	0–2	0–1	0–3
Caudal	1	0–2	0–1	0–3

D1, D2, D3, contrast medium doses (0.1 mL/kg each); 95% CI, 95% confidence intervals of the mean; Q1, first quartile; Q3, third quartile; min, minimum value; max, maximum value.

**Table 3:** Circumferential spread.

Dose	Median	95% CI	Q1–Q3	Range (min–max)
Overall circumferential spread TOT sections = 360 (*n* sections)				
D1	95	69–109	45–105	45–123
D2	43	28–53	12–52	12–60
D3	30	10–46	0–43	0–65
Thoracic circumferential spread TOT sections = 156 (*n* sections)				
D1	77	51–85	58–83	27–88
D2	22	15–28	16–28	9–32
D3	18	9–38	9–35	4–53
Lumbar circumferential spread TOT sections = 84 (*n* sections)				
D1	18	17–24	18–24	13–27
D2	17	9–25	10–23	3–31
D3	12	5–24	5–22	2–31

D1, D2, D3, contrast medium doses (0.1 mL/kg each); 95% CI, 95% confidence intervals of the mean; Q1, first quartile; Q3, third quartile; min, minimum value; max, maximum value.

**Figure 4:**
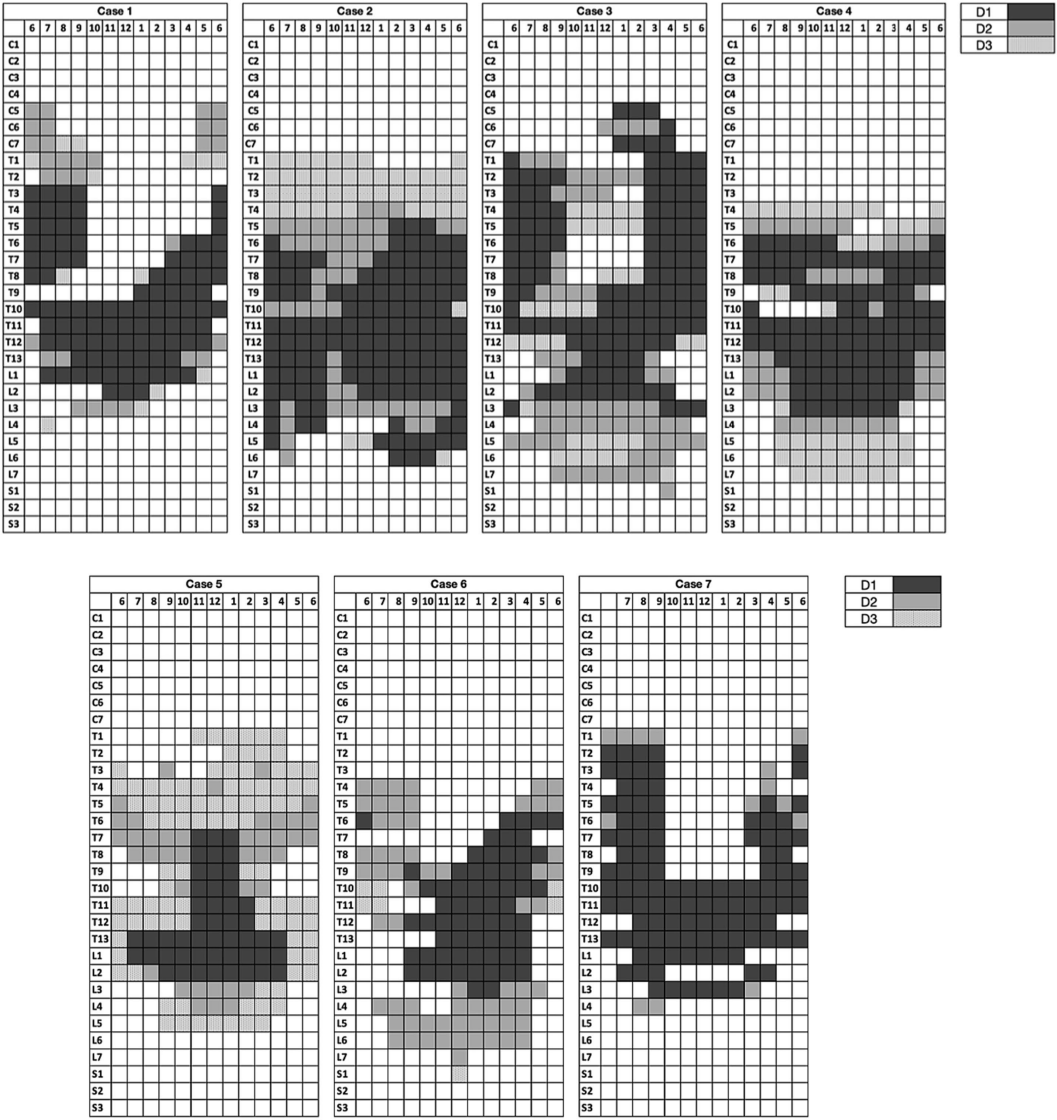
Distribution map of the contrast medium for each volume. Each row corresponds to a vertebra (at the level of the intervertebral nerve emergence) and each column represents a clock section of the spinal canal.

The distribution patterns of the medium contrast for each volume (D1, D2, and D3) for each cat are shown in Figure 4.

Cervical spread (reaching C5) was observed in two of seven cases after the injection of D1 and D2. Two out of seven cases also showed minimal circumferential sacral spread (reaching S1) after the injection of D2 and D3.

**Figure 5:**
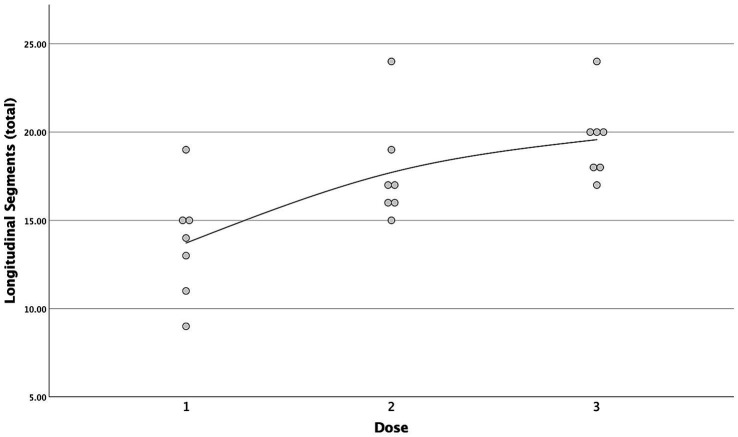
Scatterplot with interpolation line of the total longitudinal segments reached with each dose of contrast medium. Each dot represents a single case.

**Figure 6:**
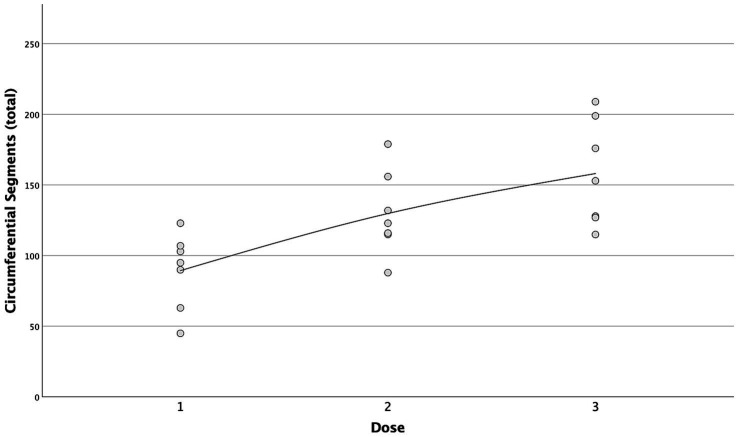
Scatterplot with interpolation line of the total circumferential segments reached with each dose of contrast medium. Each dot represents a single case.

A strong positive correlation was found between the incremental doses and both the total longitudinal and circumferential total spread (Figures 5, 6) [*τ* = 0.63 (0.4–0.78 95%CI), *p* < 0.001 and *τ* = 0.58 (0.33–0.75 95%CI), *p* = 0.001, respectively]. When the median total number of new segments reached with each aliquot of contrast medium was considered, D2 resulted in an increase of 46 and 45% for longitudinal and circumferential spread, respectively, compared to D1. For D3, this increase was of 17 and 70% for longitudinal and circumferential spread respectively, compared to D2.

**Figure 7:**
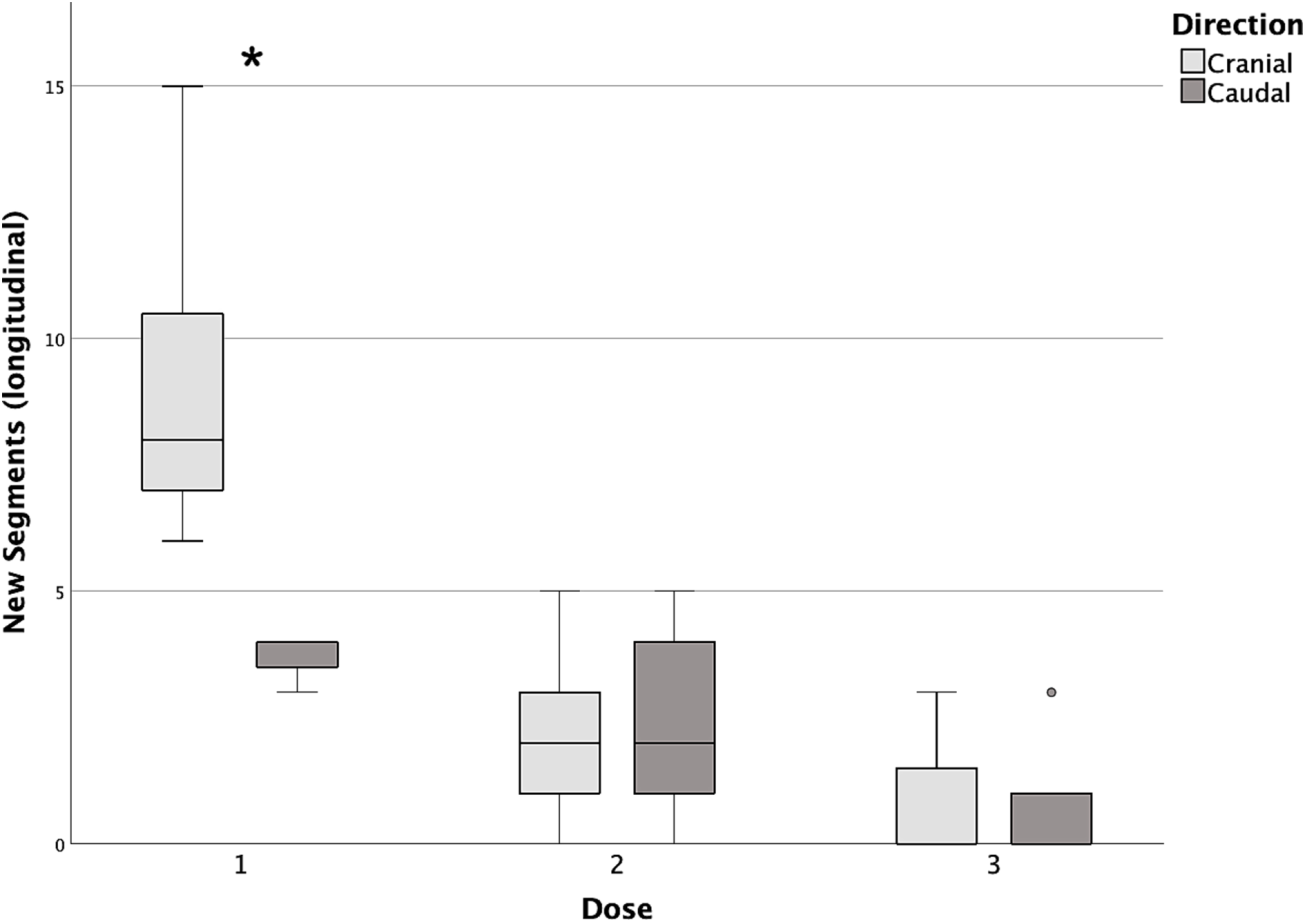
Box and whisker plots of cranial and caudal new longitudinal segments reached with each dose of contrast medium. ^*^Statistically significant difference between cranial and caudal new segments reached (*p* < 0.05).

**Figure 8:**
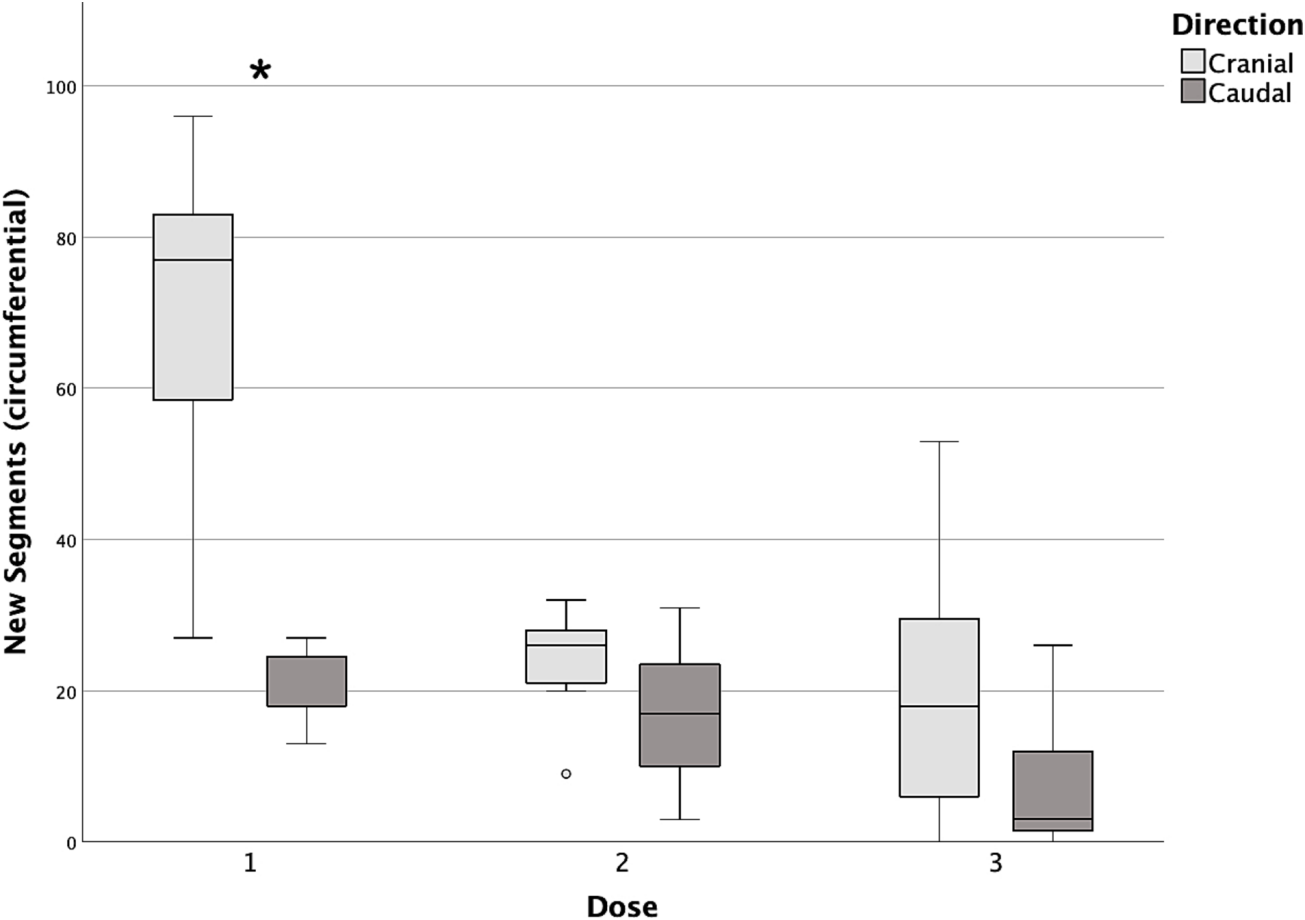
Box and whisker plots of cranial and caudal new circumferential segments reached with each dose of contrast medium. ^*^Statistically significant difference between cranial and caudal new segments reached (*p* < 0.05).

Differences in cranial and caudal new segments reached by the contrast medium with each incremental dose are reported in Table 2. The first dose was the only one showing a statistically significant difference between the cranial and caudal new segments reached, both longitudinally and circumferentially (*p* = 0.017 and *p* = 0.018, respectively), with the number of cranial segments being higher in both cases (Figures 7, 8).

Due to the lack of previously published data to use for an *a priori* sample size calculation, a post-hoc power analysis was conducted to assess the likelihood of detecting a statistically significant difference between D1 and D2 in terms of both longitudinal and circumferential cranial and caudal new segments reached. Based on the sample size used, an *α* of 0.05 and an effect size of 1.25 for the longitudinal, and 2.14 for the circumferential spread, the calculated power was 0.99 for both the differences. Effect sizes were estimated based on mean and standard deviation of each compared set of data.

## 4 Discussion

To the best of our knowledge, this was the first study describing thoracic epidural injection in cats.

The paramedian approach with a cephalad angulation, previously described in people (13) and dogs (14), resulted to be feasible in cat cadavers using a 25G 25mm Quincke needle, as the epidural space was accessed after a single attempt in the majority of the subjects.

When performing a thoracic epidural injection in a cat, several species-specific anatomical peculiarities should be considered. Firstly, the high flexibility of the axial skeleton allows for an optimal flexion of the thoracolumbar tract and stretching of the inter arcuate ligament, thus facilitating the insertion of a needle between two adjacent vertebrae. Secondly, the vertebral articular processes, main landmarks for this block, are easily palpable at the caudal thoracic level due to the reduced length of the spinous processes. Lastly, the interspinous foramen is more exposed - being less covered by the preceding vertebra than in dogs - and consequently, a lesser cephalad angulation of the needle is required to reach the epidural space (7, 16).

For this technique of needle placement, the tactile perception of piercing the interarcuate ligament and dura mater is an important feedback for the operator. For this purpose, the use of a Tuohy needle would have represented the optimal choice, at least in theory. However, to the authors’ knowledge, the smallest Tuohy needle available on the market is a 22G, 50 mm long. Considering that the epidural space was found to be approximately at a 13 mm depth from the skin surface, the use of such a needle could have resulted in a lower stability and higher mobility once inserted, thus increasing the risk of traumatizing structures within the spinal canal (17, 18). The 25G 25mm Quincke needle used in this study allowed for an easy advancement through the interspinous space and resulted to be stable after placement, despite the lower perception of the change in resistance during its insertion.

The injection of 0.1 mL of diluted contrast medium into the caudal epidural space of cat cadavers resulted in the majority of the fluid spreading cranially around the spinal cord, mostly remaining within the thoracic tract. A similar finding has been also described in humans where the higher resistance to cervical spread is due to the thoracic intumescence (1). The injection of subsequent incremental volumes demonstrated a limited capacity to achieve further cranial or caudal longitudinal, while concurrently increasing circumferential spread. Epidural spread depends on many factors, among which the difference in cross-sectional area between different tracts of the spinal canal play a pivotal role, although this variability is less in cats than in other species (19). Dural surface area is inversely correlated to longitudinal spread in humans (20), and this might have contributed somehow to our findings. However, due to the paucity of direct morphometric data on cat dural surface area, it is currently impossible to make such an assumption.

An interesting finding of this study is the lack of influence of gravity on the epidural migration of the contrast medium, as no difference in circumferential spread was observed between the left and right side. In human anesthesia, patient’s position has been shown to be one of the factors influencing local anesthetic spread within the epidural space, and its clinical relevance is also described (21).

When observing the spread maps of this cadaveric population (Figure 1), some Y-shaped patterns could be noticed. In fact, in cases 1, 3, 6, 7 the contrast medium diffused mostly dorsally along the segments caudal to the injection site, with minimal tendency to spread laterally and ventrally.

Cranially to the injection point, instead, the medium often migrated latero-ventrally, on both sides, extending longitudinally over a considerable tract, with no dorsal spread achieved at this level even following the third injection.

This Y-shaped distribution of the contrast medium injected at the level of T12–T13 has also been observed in dogs [unpublished data (22)]. Although the clinical relevance of these findings is still unknown, an uneven circumferential spread pattern around the spinal cord may result in an incomplete blockade *in vivo*.

Although this spread pattern has no clear explanation, there are several factors potentially at play.

First, the density and viscosity of contrast medium differ significantly from those of local anesthetics (23, 24), and this may result in different spread patterns. These factors are probably even more important when those fluids are injected into a narrow space, where their distribution may be influenced more by hydrostatic forces than other variables such as gravity.

Second, the time between each injection and the following CT scan may have affected the observed spread. In fact, it cannot be excluded that a longer time would have resulted in a different distribution of the contrast medium.

Third, the spread patterns observed following the sequential injection of three aliquots of fluid through the same needle may differ from those resulting from three independent injections of volumes (0.1, 0.2, and 0.3 mL/kg administered separately).

Lastly, the presence of fibrous tissue septa within the epidural space, previously uncharacterized in cats, may restrict or alter the spread of injected substances, potentially leading to segmental distribution and limited cranial spread—observed in four out of seven cats at the C7–T1 level. Similar anatomical barriers, such as the meningovertebral ligament, have been described in dogs (25) and humans (26), particularly in the cervical and lumbar regions. These structures may contribute to limited cranial spread of epidural injections and could explain issues like block failure, partial blockade or catheter displacement in humans (27, 28). While not yet studied in cats, their presence could account for the injection patterns observed in this study.

In terms of clinical applicability of the findings of the present study, there are a few limitations that should be considered. The first, and probably most important, is the use of a cadaveric model. In living animals, blood flow increases the blood volume within the epidural canal and with its rhythmic expansion and reduction can modify the local anesthetic distribution (29). Moreover, a temperature below the physiological range can alter the viscosity of the injected fluids so as tissues hydration and elasticity.

Instead of injecting three different doses into different cadavers—which would more closely simulate a single-shot clinical scenario—we administered three incremental aliquots at 5-min intervals in the same cadaver. Nonetheless, this approach may have provided insight into how a progressive increase in volume correlates with a change in spread. Additionally, it could be hypothesized that the observed distribution patterns may resemble those occurring *in vivo* when incremental boluses are administered via an epidural catheter.

Epidural anesthesia and analgesia are mainly due to the blockade of spinal nerve roots (30). Considering the spread patterns obtained in the present study, it could be argued that the inconsistent staining of the ventral aspect of the epidural space may result in a patchy or limited desensitization *in vivo*. However, all the aforementioned inherent limitations of a cadaveric study should be considered when making this assumption, as shown in human studies. Yokoyama et al. (31) observed that although there was a general correlation between the epidural spread of contrast medium and the extension of the desensitized area, the two did not match completely.

In conclusion, this study demonstrates the technical feasibility of TEA via a paramedian approach in cats and provides novel insight into epidural fluid distribution patterns.

Further in vivo studies are required to provide a more detailed description of the spread of local anesthetics and to ascertain the clinical significance of the findings of the present study.

## Data Availability

The raw data supporting the conclusions of this article will be made available by the authors, without undue reservation.
